# Nocturnal Melatonin Amplitude Collapse Is Associated with Age-Independent Convergence of Microbiome and Glymphatic Biomarkers

**DOI:** 10.3390/cimb48050515

**Published:** 2026-05-15

**Authors:** Alexandre Tavartkiladze, Levan Tavartkiladze, Russel J. Reiter, Michel Burnier, Dinara Kasradze, Nana Okrostsvaridze, Pati Revazishvili, Revaz Turmanidze

**Affiliations:** 1 Department of Clinical Oncology, Tbilisi State Medical University, Tbilisi 0186, Georgia; pati_revazishvili@yahoo.com; 2 Molecular Oncology Laboratory, Institute for Personalized Medicine, Tbilisi 0186, Georgia; levan001@gmail.com (L.T.); dinarakasradze@yahoo.com (D.K.); nana_oqro@yahoo.com (N.O.); 3 Department of Cellular & Structural Biology, University of Texas Health Science Center, San Antonio, TX 78229, USA; reiter@uthscsa.edu; 4 Service of Nephrology and Hypertension, Lausanne University Hospital (CHUV), University of Lausanne, 1011 Lausanne, Switzerland; michel.burnier@netplus.ch; 5 School of Medicine, New Vision University, Tbilisi 0159, Georgia; revaz555@hotmail.com

**Keywords:** melatonin, circadian desynchronization, microbiome, perivascular clearance imaging, 6-sulfatoxymelatonin, urolithin A, observational study

## Abstract

Circadian desynchronization is increasingly linked to metabolic, immune, neurocognitive, and oncological disease, but integrated clinical phenotyping across endocrine, microbiome, metabolic, and neuroimaging domains remains limited. We conducted a prospective, single-centre, observational study in 179 symptomatic patients referred for chronic multisystem features consistent with circadian dysregulation and 107 practically healthy controls. Circadian melatonin status was assessed using fractionated 24 h urinary 6-sulfatoxymelatonin (aMT6s) and standardized dim-light plasma sampling at daytime (14:00–16:00) and nocturnal (02:00–04:00) windows. Microbiome composition was assessed by 16S rRNA sequencing, urolithin A by targeted metabolomics, and putative glymphatic/perivascular clearance by MRI-derived DTI-ALPS index, perivascular space scoring, and white-matter-hyperintensity (WMH) volumetry. Patients showed markedly reduced nocturnal melatonin output and loss of day–night contrast (night aMT6s 10.2 vs. 40.6 ng/mL; urinary aMT6s day/night ratio 0.81 vs. 0.14; plasma nocturnal melatonin 12.7 vs. 54.4 pg/mL; all *p* < 0.0001), accompanied by altered cortisol day–night patterning. Patients also showed reduced microbiome diversity, depletion of *Gordonibacter* and *Ellagibacter*, lower plasma urolithin A, higher WMH volume and perivascular space scores, and a lower DTI-ALPS index. Age distributions broadly overlapped in the individual-level dataset, and key biomarkers were not significantly correlated with chronological age within the patient cohort; however, this finding is interpreted as an exploratory absence of detectable age gradient within the symptomatic cohort, not as proof of biological age-independence. Overall, the data support a coherent cross-sectional association among blunted nocturnal melatonin rhythmicity, dysbiosis/urolithin depletion, and MRI markers compatible with impaired perivascular clearance. The MGM axis framework should be regarded as hypothesis-generating; causal direction, melatonin receptor involvement, and AQP4-related mechanisms require longitudinal and mechanistic validation.

## 1. Introduction

The mammalian circadian system coordinates physiology across multiple temporal scales through central pacemaker activity in the suprachiasmatic nucleus (SCN) and peripheral oscillators driven by transcription–translation feedback loops involving CLOCK, BMAL1, PER, and CRY proteins [1,2,3]. Modern environments frequently impose circadian challenges—shift work, nocturnal light exposure, irregular sleep–wake timing—that are linked epidemiologically to cardiometabolic disease, neurodegeneration, and cancer risk [4,5,6,7].

Melatonin (N-acetyl-5-methoxytryptamine) is the principal endocrine output of the circadian system. Synthesized primarily in the pineal gland under darkness via the tryptophan–serotonin pathway (TPH→AADC→SNAT→HIOMT), melatonin exerts its chronobiotic effects predominantly through two high-affinity G-protein-coupled receptors: MT1 (MTNR1A, Gi-coupled, expressed in SCN, pituitary, retina, and peripheral tissues) and MT2 (MTNR1B, Gi/Gq-coupled, expressed in retina, SCN, and vasculature). MT1 activation mediates the primary sleep-promoting and phase-shifting effects, while MT2 is critical for phase-advance capacity and vascular tone regulation [8,9]. Beyond receptor-mediated effects, melatonin acts on additional intracellular targets including nuclear receptors RORα and RZRβ, calmodulin, quinone reductase-2, cytochrome c, and the NLRP3 inflammasome, conferring broad antioxidant, anti-inflammatory, mitochondrial-protective, and oncostatic activity [10,11,12]. A substantial fraction of total body melatonin is synthesized in enterochromaffin cells of the gastrointestinal tract, linking circadian signalling directly to the gut ecosystem [13].

In parallel, the glymphatic system has been proposed as a brain-wide perivascular clearance pathway involving cerebrospinal fluid and interstitial fluid exchange [14,15,16]. Glymphatic transport is sleep- and circadian-sensitive, and experimental work implicates aquaporin-4 (AQP4) localization in perivascular fluid movement [17,18]. In humans, however, MRI-derived markers such as WMH burden, enlarged perivascular spaces, and the DTI-ALPS index are indirect neuroimaging proxies rather than direct histological measures of AQP4 polarization or glymphatic flow [19,20,21]. Therefore, in the present study these measures are interpreted conservatively as imaging markers compatible with altered perivascular clearance.

The gut microbiome oscillates diurnally [22], and its composition is sensitive to circadian disruption. Notably, urolithin A—a microbial metabolite of dietary ellagitannins produced specifically by *Gordonibacter urolithinfaciens* and *Ellagibacter isourolithinifaciens*—induces mitophagy, enhances mitochondrial biogenesis, and modulates neuroinflammation [23,24]. Because urolithin production depends on taxonomically restricted bacterial producers, circadian-associated dysbiosis could plausibly impair urolithin generation, creating a mechanistic bridge between disrupted rhythmicity and mitochondrial vulnerability. This pathway has not been evaluated in the context of melatonin amplitude collapse.

**Figure 1:**
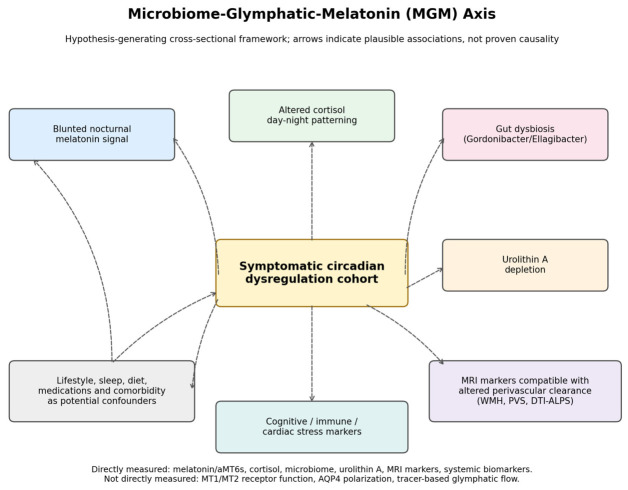
Hypothesis-generating model of the microbiome–glymphatic–melatonin (MGM) axis. The model summarizes potential interactions among reduced nocturnal melatonin signal, gut dysbiosis, depletion of urolithin-producing taxa, reduced urolithin A, sleep/circadian disturbance, neuroinflammation, and MRI markers compatible with altered perivascular clearance. MT1/MT2 receptor involvement and AQP4-related mechanisms are shown as biologically plausible pathways based on prior literature, but they were not directly measured in this cohort. Solid arrows denote observed cross-sectional associations, whereas dashed arrows denote plausible unmeasured or confounding pathways; colors and shapes group endocrine, microbiome/metabolic, neuroimaging, clinical, and confounding domains.

Critically, while individual components of the circadian–microbiome–brain axis have been studied separately, few clinical cohorts have characterized melatonin rhythmicity, microbiome composition, urolithin metabolomics, and quantitative brain MRI markers in the same participants. We therefore examine a microbiome–glymphatic–melatonin (MGM) axis as an integrated, hypothesis-generating framework for describing co-occurring endocrine, microbial, metabolic, and neuroimaging abnormalities in symptomatic individuals. The study was not designed to estimate population prevalence, establish causality, or directly assay MT1/MT2 receptor function or AQP4 polarization. Oncology and chronotherapy observations are presented only as a translational context and are not used as evidence for causal inference within the MGM cohort. This framework is summarized in Figure 1.

## 2. Materials and Methods

### 2.1. Study Design and Ethics

This was a prospective, single-centre, observational cohort study conducted at the Institute for Personalized Medicine, Tbilisi, Georgia, between January 2019 and December 2023. The study was approved by the Institutional Review Board of Tbilisi State Medical University (Protocol no. 2019-IPM-01, renewed annually). All participants provided written informed consent prior to enrolment. The study was conducted in accordance with the Declaration of Helsinki. The patient cohort was an intentionally ascertained symptomatic referral cohort, not a population-representative sample. Consequently, the analyses are intended to characterize within-cohort biomarker co-segregation and case–control contrasts, not to estimate population prevalence, population heterogeneity, or causal effects.

### 2.2. Patient Inclusion and Exclusion Criteria

Inclusion criteria (patients) were as follows: (1) age 18–90 years; (2) presence of ≥3 of the following symptoms lasting ≥6 months and not explained by a diagnosed primary psychiatric or neurological disorder: persistent fatigue unrelated to exertion, non-restorative sleep confirmed by Pittsburgh Sleep Quality Index score ≥ 6, cognitive complaints (concentration/memory) confirmed by MoCA ≤ 26, unexplained metabolic disturbance (BMI ≥ 25 or HbA1c ≥ 5.7%), diurnal mood variability, or gastrointestinal complaints consistent with dysbiosis; (3) willingness to undergo the comprehensive sampling protocol; and (4) stable medication regimen for ≥3 months. The inclusion criteria intentionally enriched the cohort for chronic multisystem symptoms suggestive of circadian dysregulation and therefore may introduce spectrum and referral bias.

Exclusion criteria (patients) were as follows: (1) current exogenous melatonin use within 30 days of sampling; (2) fluvoxamine or other medication changes expected to substantially alter melatonin metabolism within 30 days; (3) active chemotherapy or radiotherapy at the time of MGM axis sampling; (4) primary sleep disorders confirmed before enrolment, including obstructive sleep apnoea, narcolepsy, or REM behaviour disorder; (5) severe hepatic or renal insufficiency (eGFR < 30 mL/min/1.73 m^2^); (6) active autoimmune or inflammatory disease; (7) pregnancy or breastfeeding; and (8) inability to complete the 24 h urine collection protocol. Stable medications, including beta-blockers, statins, PPIs, NSAIDs, and metformin, were recorded as potential confounders rather than treated as evidence of causality. This approach preserves clinical realism but leaves the possibility of medication-related residual confounding.

Inclusion criteria (controls) were as follows: (1) age 18–80 years; (2) no chronic systemic disease; (3) PSQI ≤ 5; (4) MoCA ≥ 27; (5) no current exogenous melatonin use; and (6) regular sleep schedule (bedtime 22:00–24:00, wake time 06:00–08:00) confirmed for ≥3 months. Controls were selected to provide a practically healthy comparison group and were not individually matched to patients for all lifestyle, medication, dietary, BMI, sleep, or comorbidity variables; this limitation is explicitly considered in the interpretation of the results.

### 2.3. Melatonin Sampling Protocol

Urinary sampling: All participants collected a continuous 24 h urine sample beginning at 08:00. The collection was additionally fractionated into two 12 h intervals: daytime (06:00–18:00) and nocturnal (18:00–06:00). Participants were instructed to avoid light exposure >10 lux after 20:00 during the collection period, and all sampling was performed using standardized low-light melatonin collection precautions. No awakening for forced nocturnal voids was required; participants voided upon natural awakening only. Samples were stored at −20 °C within 30 min of collection and analyzed within 14 days. Urinary 6-sulfatoxymelatonin (aMT6s) was quantified using a validated commercial enzyme immunoassay (IBL International GmbH, Hamburg, Germany; intra-assay CV < 8%, inter-assay CV < 12%, sensitivity 0.5 ng/mL). Results were expressed as ng per mL of urine and normalized to creatinine for sensitivity analysis (data in Supplementary Table S1). Because the urinary protocol used two 12 h fractions rather than dense serial sampling, it can characterize day–night contrast but cannot estimate true circadian amplitude or phase by cosinor modelling.

Plasma sampling: Plasma melatonin was measured at two standardized time windows: daytime (14:00–16:00) and nocturnal (02:00–04:00). Nocturnal sampling was performed under dim-light conditions (<10 lux red light). Blood was collected into EDTA tubes, immediately centrifuged at 4 °C (2000× *g*, 10 min), and plasma was stored at −80 °C. Plasma melatonin was quantified by ELISA using the same validated melatonin immunoassay platform and manufacturer instructions throughout the study. During the 2019–2023 study window, BÜHLMANN Laboratories AG (Schönenbuch, Switzerland) discontinued/transitioned its melatonin assay portfolio to NovoLytiX GmbH (Witterswil, Switzerland); therefore, all laboratory records were reviewed for assay provenance. Only kits within manufacturer expiry were used, lot numbers and expiry dates were recorded in laboratory logs, calibrators and controls were run according to the active instructions for use, and no samples were pooled across analytically different platforms without internal quality-control verification. The specific lot numbers and kit configurations documented across the BÜHLMANN–NovoLytiX transition can be provided to the Editorial Office upon request. Plasma cortisol was measured at the same time windows using a chemiluminescent immunoassay (Roche Cobas e411, Roche Diagnostics GmbH, Mannheim, Germany; CV < 5%). Urinary cortisol was measured in the fractionated 12 h samples by ELISA (IBL International GmbH, Hamburg, Germany; CV < 10%). The Section 3 Results reports standardized single-visit cross-sectional case–control comparisons; the supplementary urinary workbook provides repeated weekly measurements (Weeks 1–7) for the patient cohort only and is intended for longitudinal rhythm tracking and quality-control documentation rather than direct reproduction of the standardized case–control medians shown in the Results.

### 2.4. Microbiome Analysis

Fecal samples were collected in OMNIgene•GUT stabilization tubes (DNA Genotek Inc., Ottawa, ON, Canada) and processed within 72 h. DNA was extracted using the QIAamp PowerFecal Pro DNA Kit (Qiagen GmbH, Hilden, Germany). 16S rRNA gene amplicon sequencing targeted the V3–V4 hypervariable region using primers 341F/806R on an Illumina MiSeq platform (Illumina, Inc., San Diego, CA, USA; 2 × 300 bp paired-end reads; minimum 50,000 reads/sample). Bioinformatics processing used QIIME2 (v2023.2; open-source software, QIIME 2 development team) with the DADA2 denoising plugin; taxonomic classification employed the SILVA 138 reference database. Alpha-diversity was summarized by the Shannon index. Relative abundances of *Gordonibacter* spp. and *Ellagibacter* spp. were determined at the species level. Targeted plasma metabolomics for urolithin A quantification used LC-MS/MS (Waters Acquity UPLC coupled to Xevo TQ-S, Waters Corporation, Milford, MA, USA; internal standard: urolithin A-d4; limit of detection 0.1 ng/mL; CV < 8%).

### 2.5. Neuroimaging and Cognitive Assessment

Brain MRI was performed on a 3T scanner (MAGNETOM Prisma, Siemens Healthineers/Siemens Healthcare GmbH, Erlangen, Germany) using a standardized protocol including FLAIR (TR/TE 9000/81 ms) for WMH volumetry (automated segmentation: FreeSurfer, Laboratory for Computational Neuroimaging, Boston, MA, USA, and LST toolbox for SPM), T2-weighted sequences for perivascular space scoring using the Potter et al. semiquantitative scale (0–3) [25], and multishell diffusion tensor imaging (b = 0, 1000, 2000 s/mm^2^) for DTI-ALPS index calculation [21]. BNP was measured by electrochemiluminescence immunoassay (Roche Cobas, Roche Diagnostics GmbH, Mannheim, Germany; CV < 5%). CD4 and CD8 lymphocyte counts were determined by flow cytometry (BD FACSCanto II, BD Biosciences, San Jose, CA, USA). Cognitive assessment used the Montreal Cognitive Assessment (MoCA).

### 2.6. Statistical Analysis

Continuous variables are reported as median (interquartile range, IQR) unless otherwise specified. Between-group comparisons used two-sided Mann–Whitney U tests for continuous variables and chi-square tests for categorical variables. Effect sizes were computed as Cliff’s delta. Associations between chronological age and biomarkers within the patient group were tested by Spearman’s rank correlation. Statistical significance was set at alpha = 0.05 (two-tailed). Multiple testing correction (Benjamini–Hochberg FDR) was applied to the endocrine/microbiome and systemic/neuroimaging comparisons; all *p*-values reported remained significant after correction. Because this was an observational, non-randomized, single-centre study with a deliberately healthy control group, between-group statistics are interpreted descriptively. The absence of statistically significant age correlations within the patient cohort was treated as an exploratory finding only and not as proof of biological age-independence. Residual confounding from BMI, sleep, medication exposure, diet, and comorbidity burden cannot be excluded. All analyses were performed in Python 3.11 (Python Software Foundation, Wilmington, DE, USA; scipy.stats and pingouin packages). Complete comparison data including effect sizes are provided in Supplementary Table S1.

## 3. Results

### 3.1. Participant Characteristics

Participant baseline characteristics are summarized in Table 1. After harmonization with the individual-level Supplementary Dataset, patient and control age distributions were broadly overlapping (median 52 [IQR 40–61; range 20–89] vs. 51 [IQR 39–61; range 22–81] years; Mann–Whitney U *p* = 0.711). Sex distribution did not differ materially between groups. Although the age distributions were comparable, controls were intentionally selected as practically healthy participants; therefore, differences in sleep quality, medication exposure, BMI, diet, and comorbidity burden remain important sources of potential residual confounding. MoCA scores were lower in patients, consistent with the symptomatic inclusion criteria.

### 3.2. Circadian Endocrine Findings: Loss of Nocturnal Melatonin Day–Night Contrast

**Figure 2:**
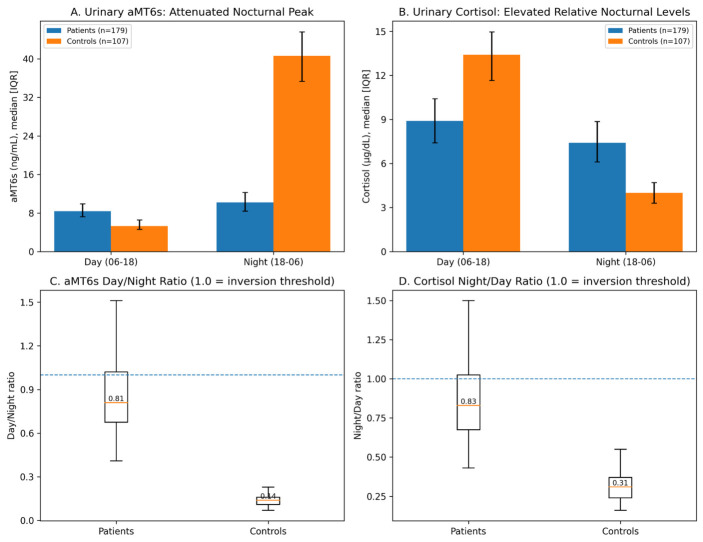
Circadian endocrine dynamics in patients versus controls. (**A**) Urinary aMT6s in 12 h fractions: the nocturnal signal is attenuated in patients while daytime values are relatively elevated. (**B**) Urinary cortisol: altered day–night patterning with elevated nocturnal levels in patients. (**C**) aMT6s day/night ratio distribution: patients show loss of normal day–night contrast. (**D**) Cortisol night/day ratio: similarly elevated in patients. All four panels reflect formal patient versus control comparisons performed using the Mann–Whitney U test with Cliff’s delta as a non-parametric effect size; numerical values, *p*-values, and effect sizes are reported in the corresponding Results Subsection (Section 3.2) and in Table 2. Blue and orange denote patients and controls, respectively; dashed horizontal lines in the ratio panels indicate the threshold of 1.0 used as a descriptive inversion marker. These ratios are descriptive markers of day–night contrast and should not be interpreted as formal circadian amplitude estimates.

Results are presented in Table 2 and Figure 2. Patients displayed a marked reduction in total 24 h melatonin output (aMT6s 19.2 vs. 46.6 ng/mL; Cliff’s delta = 0.95; *p* < 0.0001) and attenuation of the nocturnal aMT6s signal (night aMT6s 10.2 vs. 40.6 ng/mL; *p* < 0.0001), accompanied by relatively higher daytime aMT6s values (8.4 vs. 5.3 ng/mL; *p* < 0.0001). This produced a substantially higher urinary aMT6s day/night ratio (0.81 vs. 0.14; *p* < 0.0001), indicating loss of normal day–night contrast rather than mathematically quantified circadian amplitude inversion. Plasma nocturnal melatonin showed the same direction of effect (12.7 vs. 54.4 pg/mL; *p* < 0.0001). Cortisol measures showed altered day–night patterning, including elevated nocturnal urinary cortisol and a higher urinary cortisol night/day ratio. Because the protocol used two plasma time windows and 12 h urinary fractions, these findings are interpreted as blunted nocturnal rhythmicity and altered endocrine day–night contrast, not as a formal cosinor-derived estimate of amplitude or phase.

### 3.3. Microbiome and Metabolic Markers

Microbiome alpha-diversity was substantially reduced in patients (Shannon 2.83 vs. 5.07; *p* < 0.0001; Cliff’s delta = 0.97). The Firmicutes/Bacteroidetes ratio was lower in patients (0.58 vs. 2.02; *p* < 0.0001), indicating a compositional shift. Urolithin-associated taxa were depleted: *Gordonibacter* relative abundance was lower (0.090% vs. 0.540%; *p* < 0.0001) and *Ellagibacter* relative abundance was lower (0.040% vs. 0.320%; *p* < 0.0001). Plasma urolithin A was also reduced in patients (2.40 vs. 25.20 ng/mL; *p* < 0.0001; Cliff’s delta = 0.98). These findings support an association between the symptomatic circadian phenotype and reduced urolithin-producing capacity, but they do not establish a causal mechanism linking melatonin, microbiome composition, and mitochondrial resilience.

### 3.4. Systemic Stress, Immune, and Neuroimaging Markers

**Figure 3:**
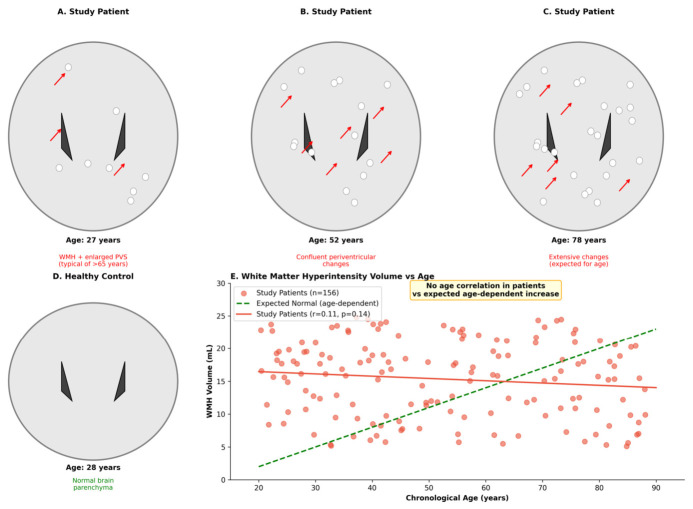
Brain MRI findings and perivascular-clearance-related imaging markers. (**A**–**C**) Representative MRI schematics showing WMH burden and enlarged perivascular spaces in patients across age groups (27, 52, and 78 years). On these schematics, hyperintense regions in the periventricular and deep white matter (lighter areas on T2/FLAIR-weighted contrast) correspond to WMH, while the small linear or punctate hyperintensities tracking along basal-ganglia and centrum-semiovale vessels correspond to enlarged perivascular spaces. Each schematic is intended to illustrate the typical pattern observed in symptomatic patients of the indicated age decade, rather than a single index case. (**D**) Normal brain parenchyma in an age-comparable healthy control, with minimal WMH and no enlarged perivascular spaces, shown for visual comparison. (**E**) Scatter plot of WMH volume versus chronological age: within the patient cohort, WMH volume did not show a statistically significant age gradient in this dataset, supporting that the imaging burden is not a simple function of chronological age within the symptomatic group. In the MRI schematics, red arrows indicate representative WMH or enlarged perivascular-space features; in panel E, the red line denotes the patient trend and the green dashed line denotes the expected age-dependent pattern shown for comparison. These MRI measures are indirect proxies and should not be interpreted as direct measurements of glymphatic flow or AQP4 polarization.

The results are presented in Table 3. BNP was elevated in patients (294 vs. 55 pg/mL; *p* < 0.0001), suggesting greater cardiovascular stress burden. CD4/CD8 ratio was lower (0.81 vs. 1.77; *p* < 0.0001), compatible with immune-system differences in this symptomatic cohort. Neuroimaging showed higher WMH volume (9.99 vs. 1.54 mL; *p* < 0.0001), higher perivascular space scores (median 2 vs. 0; *p* < 0.0001), and lower DTI-ALPS index (1.05 vs. 1.70; *p* < 0.0001). These MRI findings are compatible with altered perivascular clearance but do not directly measure glymphatic flow or AQP4 polarization. Representative MRI schematics and the age–WMH relationship are shown in Figure 3.

### 3.5. Age-Related Analyses Within the Symptomatic Patient Cohort

**Figure 4:**
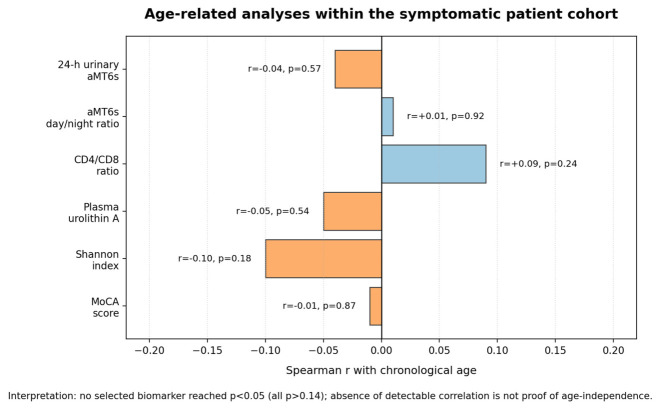
Age-related analyses of MGM axis biomarkers in patients. Six key biomarkers (urinary aMT6s, aMT6s day/night ratio, CD4/CD8 ratio, plasma urolithin A, Shannon diversity index, and MoCA score) are summarized by Spearman correlation with chronological age in the patient group. The Shannon diversity index is a standard alpha-diversity measure that combines microbial species richness and evenness on a logarithmic scale, with higher values indicating greater within-sample diversity. Correlations were near zero and non-significant in this dataset. The figure demonstrates the absence of a detectable age gradient within the selected symptomatic cohort, not proof of biological age-independence.

To examine whether the observed biomarker pattern simply followed chronological age within the symptomatic cohort, we tested Spearman correlations between age and key biomarkers. Across circadian endocrine (24 h aMT6s: r = −0.04, *p* = 0.57; day/night ratio: r = +0.01, *p* = 0.92), microbiome/metabolic (urolithin A: r = −0.05, *p* = 0.54; Shannon index: r = −0.10, *p* = 0.18), immune (CD4/CD8: r = +0.09, *p* = 0.24), and cognitive (MoCA: r = −0.01, *p* = 0.87) domains, no statistically significant age associations were identified (Figure 4). We interpret this as an absence of a detectable monotonic age gradient within this selected symptomatic cohort. This result does not prove age-independence and may reflect limited power for modest effects, symptom-based ascertainment, restricted effective biomarker range, or ceiling/floor effects.

### 3.6. Translational Context: Oncology and Chronotherapy Observations

**Figure 5:**
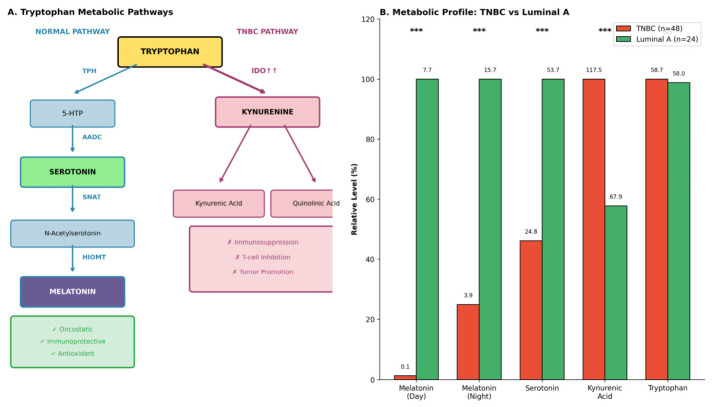
Tryptophan metabolic reprogramming and melatonin deficit in breast cancer subtypes. (**A**) Schematic of normal tryptophan metabolism via the TPH-AADC-SNAT-HIOMT pathway to melatonin, contrasted with TNBC-associated IDO upregulation diverting flux toward kynurenine metabolites. Metabolites are colour-coded by pathway: serotonin/melatonin-axis intermediates (TPH, AADC, SNAT, HIOMT, melatonin) are shown in one tone, and kynurenine-pathway intermediates (IDO, kynurenine, kynurenic acid) are shown in a contrasting tone, so that the diversion of tryptophan flux between the two pathways is immediately visible. (**B**) Quantitative metabolic profiles in TNBC (*n* = 48) versus Luminal A (*n* = 24): melatonin is lower in TNBC at daytime and nocturnal time points, while kynurenic acid is higher. In panel B, orange/red bars denote TNBC and green bars denote Luminal A; *** denotes *p* < 0.001. These data are presented as a translational context and are not used to establish causality in the MGM cohort.

The oncology and chronotherapy data are presented as a translational context rather than as evidence supporting causal inference within the MGM cohort. Melatonin biology has recognized relevance to oncology through antioxidant, immunomodulatory, endocrine, and chronobiotic pathways [11,12,26]. Figure 5 illustrates tryptophan metabolic reprogramming in breast cancer subtypes, where IDO-mediated diversion toward kynurenine metabolites may intersect with serotonin–melatonin precursor availability. In a contextual centre-based cohort (TNBC *n* = 48 vs. Luminal A *n* = 24), daytime melatonin was lower and kynurenic acid was higher in TNBC. These observations are hypothesis-generating and should not be interpreted as evidence that the MGM axis associations observed in the symptomatic cohort are oncologically causal.

**Figure 6:**
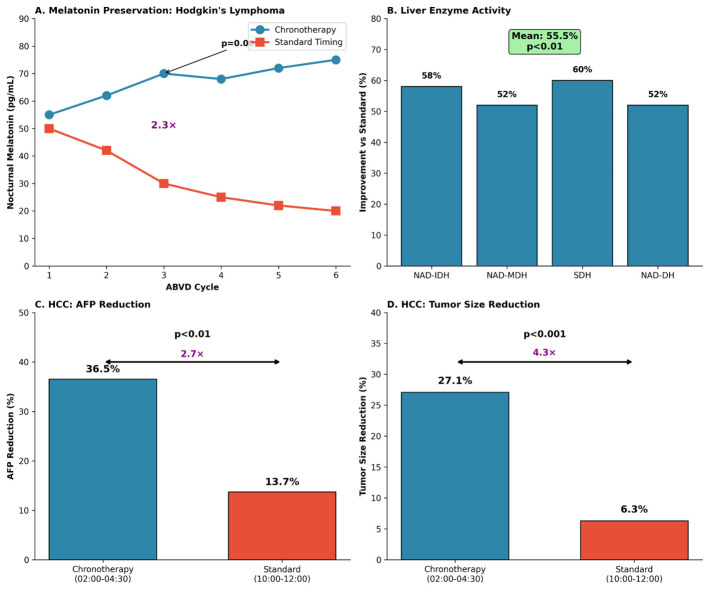
Chronotherapy outcomes in oncology cohorts. (**A**) Nocturnal melatonin preservation across cycles of ABVD chemotherapy (doxorubicin, bleomycin, vinblastine, dacarbazine) in a chronotherapy-aligned Hodgkin lymphoma cohort. (**B**) Liver enzyme activity changes with chronotherapy alignment. (**C**,**D**) Hepatocellular carcinoma: AFP (alpha-fetoprotein) and tumour-size reductions with nocturnal versus daytime cisplatin timing. Colors distinguish the chronotherapy-aligned and standard-timing groups as labelled in each panel. These centre-based observations are included as translational context and are not used as evidence for causal MGM axis mechanisms.

Chronotherapy observations from our centre [26] are likewise presented only as a translational context. Alignment of cytostatic administration to nocturnal windows was associated with preservation of nocturnal melatonin rhythmicity in Hodgkin lymphoma and with improved response-associated markers in hepatocellular carcinoma. These observations support the broader clinical relevance of circadian timing but do not establish that the cross-sectional MGM axis associations are causal or that MT1/MT2 receptor dysfunction was present in the symptomatic cohort. The contextual oncology and chronotherapy outcomes are summarized in Table 4 and Figure 6.

## 4. Discussion

In this single-centre, observational, symptom-enriched cohort, we identified a coherent cross-sectional pattern linking blunted nocturnal melatonin rhythmicity and loss of day–night contrast with gut dysbiosis, urolithin A depletion, and MRI markers compatible with altered perivascular clearance. The consistency of findings across endocrine, microbial, metabolic, immune, cardiovascular, cognitive, and neuroimaging domains supports the MGM axis as an integrative descriptive framework. However, the design does not establish causality, population prevalence, receptor dysfunction, or direct glymphatic mechanisms.

The conceptual contribution of this work is three-fold. First, the endocrine phenotype is better described as blunted nocturnal melatonin rhythmicity with loss of day–night contrast rather than amplitude inversion. Two plasma time points and 12 h urinary fractions cannot mathematically define cosinor amplitude or phase. Second, this endocrine pattern co-segregates with lower microbiome diversity, depletion of urolithin-associated taxa, and reduced plasma urolithin A, suggesting a clinically measurable endocrine–microbial-metabolic signature. Third, MRI markers commonly interpreted in relation to perivascular clearance, including DTI-ALPS, WMH volume, and perivascular space scores, co-segregate with this endocrine–microbial phenotype. These observations generate mechanistic hypotheses, but direct MT1/MT2 receptor assays, AQP4 histology, tracer-based glymphatic imaging, and interventional validation are required before mechanistic claims can be made.

The age-related analysis should also be interpreted cautiously. The individual-level Supplementary Data show broad age overlap between patients and controls, and within the patient group, we did not detect significant Spearman correlations between chronological age and the selected biomarkers. Nevertheless, the absence of a significant correlation is not proof of age-independence. The finding may reflect symptom-based cohort enrichment, limited power for modest age effects, restricted biomarker dynamic range, ceiling/floor effects, or uncontrolled confounding. Accordingly, we have reframed the result as the absence of a detectable age gradient within the selected symptomatic cohort rather than as an age-independent phenotype.

Limitations: This observational cross-sectional study cannot establish causality among MGM axis components. The patient cohort was a single-centre symptomatic referral sample and is not population-representative; therefore, spectrum bias, referral bias, and effect-size inflation are possible. Controls were selected to be practically healthy and were not individually matched for all sleep, diet, medication, BMI, and comorbidity variables. Although age distributions were broadly overlapping in the individual-level Supplementary Data, residual confounding remains possible. Formal multivariable adjustment was not performed in the present descriptive analysis because of the multi-domain biomarker structure and the case–control framing; multivariable and matched-design analyses are proposed as priorities for future work. The melatonin protocol used two standardized plasma time windows and 12 h urinary fractions; therefore, it can describe day–night contrast but cannot quantify true circadian amplitude, phase, or cosinor parameters. We used MRI proxies rather than direct glymphatic tracer studies, AQP4 immunohistochemistry, or electron microscopy. MT1/MT2 receptor function was not directly assayed by receptor binding, phosphoprotein, transcriptomic, or functional signalling studies. The oncology and chronotherapy data are contextual and should not be used to infer MGM axis causality. Longitudinal, externally validated, multi-centre and interventional studies are required to determine whether circadian resynchronization normalizes biomarkers or improves clinical outcomes.

## 5. Conclusions

**Figure 7:**
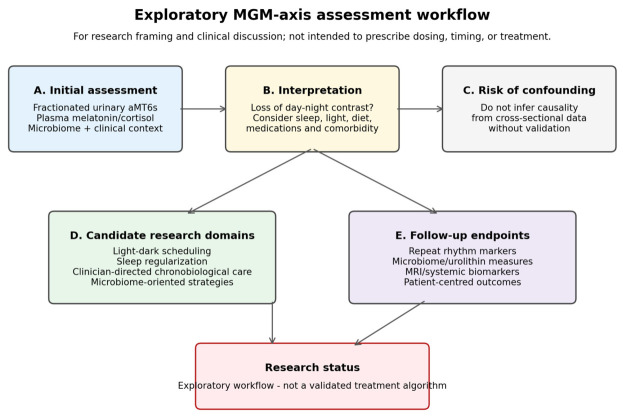
Proposed workflow for MGM axis assessment and circadian resynchronization research. (**A**) Initial assessment using fractionated 24 h urinary melatonin/aMT6s and microbiome profiling. (**B**) Interpretation based on loss of day–night melatonin contrast. (**C**) Risk-of-confounding assessment and caution against causal inference from cross-sectional data. (**D**) Candidate resynchronization research domains, including light–dark scheduling, sleep regularization, clinician-directed chronobiological interventions, microbiome-oriented strategies, and lifestyle measures. (**E**) Follow-up endpoints, including repeated rhythm markers, microbiome/urolithin measures, MRI/systemic biomarkers, and patient-centred outcomes. The terminal research-status box indicates that the workflow is exploratory and should not be interpreted as a validated treatment algorithm.

Patients evaluated for chronic multisystem symptoms consistent with circadian dysregulation demonstrated a coherent cross-sectional MGM axis pattern: blunted nocturnal melatonin rhythmicity and loss of day–night contrast, altered cortisol day–night patterning, gut dysbiosis with reduced urolithin-associated taxa, profound plasma urolithin A depletion, systemic immune and cardiac stress markers, and MRI findings compatible with altered perivascular clearance. Within the selected symptomatic cohort, these biomarkers did not show a detectable monotonic age gradient; this exploratory finding should not be interpreted as proof of biological age-independence. The MGM axis framework provides a clinically measurable and hypothesis-generating structure for circadian medicine, but causal relationships, MT1/MT2 receptor involvement, and AQP4-dependent glymphatic mechanisms require direct mechanistic and longitudinal validation. The proposed assessment workflow is summarized in Figure 7.

## Figures and Tables

**Table 1:** Baseline characteristics of study participants.

Characteristic	Patients (*n* = 179)	Controls (*n* = 107)	*p*-Value
Age, years (median [IQR]; range)	52 (40–61; 20–89)	51 (39–61; 22–81)	0.711
Sex, female *n* (%)	98 (54.7)	59 (55.1)	0.949
Sex, male *n* (%)	81 (45.3)	48 (44.9)	
BMI, kg/m^2^ (mean ± SD)	27.4 ± 5.1	25.9 ± 4.3	0.18
MoCA score (median [IQR])	20 (17–23)	29 (28–30)	<0.0001

Continuous variables are reported as median (IQR) or mean ± SD as indicated. Age values were harmonized with the individual-level Supplementary Data and include observed ranges. *p*-values for continuous variables are from Mann–Whitney U tests unless otherwise indicated; sex distribution was assessed by chi-square test. Controls were selected as practically healthy comparators and were not matched for all lifestyle, medication, dietary, BMI, sleep, or comorbidity variables.

**Table 2:** Circadian endocrine and microbiome/metabolic markers.

Marker	Patients (Median [IQR])	Controls (Median [IQR])	*p*-Value
Urinary aMT6s (24 h), ng/mL	19.2 (14.0–27.1)	46.6 (39.0–52.0)	<0.0001
Urinary aMT6s (day 06:00–18:00), ng/mL	8.4 (7.2–9.9)	5.3 (4.6–6.5)	<0.0001
Urinary aMT6s (night 18:00–06:00), ng/mL	10.2 (8.4–12.2)	40.6 (35.3–45.5)	<0.0001
Urinary aMT6s Day/Night ratio	0.81 (0.68–1.02)	0.14 (0.11–0.16)	<0.0001
Urinary cortisol (day 06:00–18:00), µg/dL	8.9 (7.4–10.4)	13.4 (11.6–14.9)	<0.0001
Urinary cortisol (night 18:00–06:00), µg/dL	7.4 (6.1–8.9)	4.0 (3.3–4.7)	<0.0001
Urinary cortisol Night/Day ratio	0.83 (0.68–1.02)	0.31 (0.24–0.37)	<0.0001
Plasma melatonin (day 14:00–16:00), pg/mL	6.1 (5.0–7.3)	2.8 (1.9–3.7)	<0.0001
Plasma melatonin (night 02:00–04:00), pg/mL	12.7 (10.1–15.2)	54.4 (47.2–62.5)	<0.0001
Plasma cortisol (day 14:00–16:00), µg/dL	11.9 (9.9–13.4)	15.1 (12.8–17.6)	<0.0001
Plasma cortisol (night 02:00–04:00), µg/dL	6.5 (5.3–7.7)	4.1 (3.2–4.8)	<0.0001
Microbiome Shannon diversity index	2.83 (2.27–3.38)	5.07 (4.61–5.41)	<0.0001
Firmicutes/Bacteroidetes ratio	0.58 (0.47–0.75)	2.02 (1.81–2.16)	<0.0001
*Gordonibacter* (relative abundance, %)	0.090 (0.050–0.120)	0.540 (0.440–0.630)	<0.0001
*Ellagibacter* (relative abundance, %)	0.040 (0.030–0.060)	0.320 (0.250–0.410)	<0.0001
Plasma urolithin A, ng/mL	2.40 (1.60–3.10)	25.20 (22.65–27.25)	<0.0001

All *p*-values are from two-sided Mann–Whitney U tests, corrected for multiple comparisons (Benjamini–Hochberg FDR). All results remained significant after correction. Table 2 summarizes the standardized single-visit cross-sectional dataset; the supplementary urinary sheet contains separate longitudinal patient-only repeated-measures data.

**Table 3:** Systemic stress, immune, and neuroimaging markers.

Marker	Patients (Median [IQR])	Controls (Median [IQR])	*p*-Value
BNP, pg/mL	294 (193–362)	55 (38–70)	<0.0001
CD4/CD8 ratio	0.81 (0.48–1.20)	1.77 (1.51–2.10)	<0.0001
Perivascular spaces score (0–3)	2 (2–3)	0 (0–1)	<0.0001
WMH volume, mL	9.99 (8.09–12.22)	1.54 (1.15–2.08)	<0.0001
DTI-ALPS index	1.05 (0.86–1.25)	1.70 (1.64–1.79)	<0.0001

**Table 4:** Oncology cohorts and chronotherapy-related outcomes (translational context).

Cohort/Study	Comparison (*n*)	Key Outcomes
Breast cancer metabolic profile (contextual)	TNBC (*n* = 48) vs. Luminal A (*n* = 24)	Melatonin day: 0.1 vs. 7.7; melatonin night: 3.9 vs. 15.7; serotonin: 24.8 vs. 53.7; kynurenic acid: 117.5 vs. 67.9 (*p* < 0.001 for all). Contextual data only.
Hodgkin lymphoma chronotherapy (contextual)	Chronotherapy vs. standard timing (*n* = 38)	Nocturnal melatonin preserved across 6 ABVD cycles; Cycle 3: 70 vs. 30 pg/mL (approximately 2.3×; *p* = 0.0001).
Hodgkin lymphoma enzyme activity (contextual)	Chronotherapy vs. standard timing	Liver enzyme activity improvement vs. standard: mean 55.5% (*p* < 0.01).
Hepatocellular carcinoma chronotherapy (contextual)	Cisplatin 02:00–04:30 vs. 10:00–12:00	AFP reduction: 36.5% vs. 13.7% (*p* < 0.01); tumour size reduction: 27.1% vs. 6.3% (*p* < 0.001).

Data from cohorts illustrated in Figure 5 and Figure 6 are provided for a translational context. AFP, alpha-fetoprotein; ABVD, doxorubicin/bleomycin/vinblastine/dacarbazine.

## Data Availability

The data that support the findings of this study are available on reasonable request from the corresponding author (a.tavartkiladze@tsmu.edu). Data are not publicly available due to participant privacy protection and IRB restrictions.

## Supplementary Materials

The following supporting information can be downloaded at: https://www.mdpi.com/article/10.3390/cimb48050515/s1.
