# Supplementary material for: Nocturnal Melatonin Amplitude Collapse Is Associated with Age-Independent Convergence of Microbiome and Glymphatic Biomarkers
Source: Curr Issues Mol Biol. 2026 May 15;48(5):515. doi: 10.3390/cimb48050515 (PMC13204537; doi:10.3390/cimb48050515)
Supplement: Supplementary file 1 [file cimb-48-00515-s001.zip › Supplementary_Table_S1_Final.pdf]

Supplementary Table S1. Diagnosis categories and data completeness for the analytic cohorts.

Table S1A. Diagnosis category distribution within the chronobiology cohort (n=179).

Diagnosis category	n (%)	Age, years (mean ± SD)	Female, n (%)	aMT6s below LOD, n (%)
Solid malignancy	72 (40.2%)	53.5 ± 13.9	41 (56.9%)	7 (9.7%)
Metabolic syndrome	48 (26.8%)	48.0 ± 16.9	26 (54.2%)	6 (12.5%)
Hematologic malignancy	22 (12.3%)	53.7 ± 20.6	11 (50.0%)	1 (4.5%)
Cardiovascular	19 (10.6%)	49.2 ± 18.1	16 (84.2%)	2 (10.5%)
Neurodegenerative	18 (10.1%)	49.7 ± 13.6	8 (44.4%)	2 (11.1%)

Table S1B. Data availability and missingness by cohort.

Data element	Chronobiology cohort (n=179)	Practically healthy controls (n=107)
7-week fractionated urine (aMT6s + cortisol)	179/179 (100.0%)	Not collected
Plasma biomarker panel	179/179 (100.0%)	107/107 (100.0%)
Microbiome 16S panel	179/179 (100.0%)	107/107 (100.0%)
Medications & exposures questionnaire	179/179 (100.0%)	107/107 (100.0%)
Brain MRI / glymphatic imaging dataset	156/179 (87.2%)	80/107 (74.8%)
OCP/HRT variable recorded (women only)	49/102 (48.0%)	30/57 (52.6%)

Table S1C. Circadian endocrine measures by diagnosis category within the chronobiology cohort (n=179).

Panel S1. aMT6s (6-sulfatoxymelatonin) from fractionated urine.

Diagnosis category	n	Mean aMT6s Day (ng/mL)	Mean aMT6s Night (ng/mL)	Day/Night ratio
Solid malignancy	72	2.92 ± 0.98	7.45 ± 2.52	0.42 ± 0.10
Metabolic syndrome	48	2.84 ± 1.11	7.21 ± 2.77	0.42 ± 0.11
Hematologic malignancy	22	3.06 ± 0.73	7.71 ± 1.86	0.40 ± 0.06
Cardiovascular	19	2.97 ± 1.06	7.31 ± 2.59	0.42 ± 0.07
Neurodegenerative	18	2.89 ± 1.04	7.72 ± 2.92	0.41 ± 0.11
**Overall**	**179**	**2.92 ± 1.00**	**7.43 ± 2.55**	**0.42 ± 0.10**

Panel S2. Urinary cortisol from fractionated urine.

Diagnosis category	n	Mean cortisol Day (µg/dL)	Mean cortisol Night (µg/dL)	Night/Day ratio
Solid malignancy	72	18.62 ± 1.90	4.77 ± 0.59	0.26 ± 0.04
Metabolic syndrome	48	18.66 ± 2.50	4.76 ± 0.60	0.26 ± 0.05
Hematologic malignancy	22	18.31 ± 1.97	4.68 ± 0.59	0.26 ± 0.05
Cardiovascular	19	18.27 ± 2.07	4.73 ± 0.49	0.26 ± 0.04
Neurodegenerative	18	17.90 ± 2.19	4.77 ± 0.56	0.27 ± 0.04
**Overall**	**179**	**18.48 ± 2.12**	**4.75 ± 0.58**	**0.26 ± 0.04**

Notes: aMT6s = 6-sulfatoxymelatonin. LOD = limit of detection. Diagnosis categories and endocrine measures were derived from the 7-week fractionated urine dataset (n=179). For Table S1C, values are mean ± SD of participant-level means computed across the 7 weekly collections; daytime window = 06:00–18:00 and nighttime window = 18:00–06:00. The aMT6s panel reports Day/Night ratio; the cortisol panel reports Night/Day ratio, consistent with the expected direction of the respective endocrine rhythms and with the supplementary workbook variable dictionary. The MRI dataset was available for a subset of participants; values in Table S1B reflect availability (not within-table missingness). OCP/HRT recording is summarized among women only; missing entries may reflect either non-response or non-applicability depending on study procedures.
